# Synthesis of Micelles Guided Magnetite (Fe_3_O_4_) Hollow Spheres and their application for AC Magnetic Field Responsive Drug Release

**DOI:** 10.1038/srep35721

**Published:** 2016-10-31

**Authors:** Madhuri Mandal Goswami

**Affiliations:** 1 Dept. of Condensed Matter Physics and Material Science, S N Bose National Centre for Basic Sciences, Block JD, sector III, Salt lake, Kolkata 700098, India

## Abstract

This paper reports on synthesis of hollow spheres of magnetite, guided by micelles and their application in drug release by the stimulus responsive technique. Here oleyelamine micelles are used as the core substance for the formation of magnetite nano hollow spheres (NHS). Diameter and shell thickness of NHS have been changed by changing concentration of the micelles. Mechanism of NHS formation has been established by investigating the aliquot collected at different time during the synthesis of NHS. It has been observed that oleyelamine as micelles play an important role to generate hollow-sphere particles of different diameter and thickness just by varying its amount. Structural analysis was done by XRD measurement and morphological measurements, SEM and TEM were performed to confirm the shape and size of the NHS. FTIR measurement support the formation of magnetite phase too. Frequency dependent AC magnetic measurements and AC magnetic field stimulated drug release event by these particles provide a direction of the promising application of these NHS for better cancer treatment in near future. Being hollow & porous in structure and magnetic in nature, such materials will also be useful in other applications such as in removal of toxic materials, magnetic separation etc.

Recently magnetic nanoparticles especially with tunable size, shape and porosity are getting very importance and attraction in research^1^,^2^,^3^,^4^,^5^,^6^,^7^. It has been observed that depending on size, composition, structure of the particles, the magnetic properties are altered and from these phenomena it is presumed that magnetic particles with tuned properties may have diverse range of useful applications from magnetic resonance imaging, magnetic separation, magnetic field triggered drug delivery and release, hyperthermia therapy etc.^8^,^9^. From the practical point of view, the magnetic property of magnetic nanoparticles is a very promising application tool in cancer diagnosis and treatment. Its research involves proper design, synthesis, engineering and characterization of a wide variety of magnetic nanoparticles. It is known that the cancer drugs can not selectively target the cancer affected region, it spreads all over the body damaging the benign cells too. So treatment of cancer without damaging the healthy cells with minimal side effect is one of the goals of clinical community. Cancer drugs themselves are very harmful to body. If spreading of such drugs all over body can be controlled that will be a real benefit to cancer patients. Recently, there has been an increasing drive to search for therapeutic agents which are deliverable to a specific diseased site with aim to improve their efficiency and minimize undesirable side effects^10^,^11^. Among others, magnetic field directed targeting is one of the very promising techniques and different types of magnetic particles can be used as targeting agent. Magnetite nanoparticles are widely considered as a promising agent for the same due to its various advantageous features such as bio-safety, biocompatibility, ease of preparation and handling, low cost, stability and more importantly, possibility of carrying the drug to the desired location within the host body by using an external magnetic field. In this way, proper delivery of drugs to selective region and magnetic field stimulated drug release may provide a new and effective pathway for treatment of cancer. Now a days, different stimuli like electric field, heat, pH, light, enzymes, etc.^12^,^13^,^14^,^15^,^16^,^17^,^18^,^19^,^20^,^21^,^22^ have been used to check the drug release activity of different suitable nano drug delivery systems. Drug delivery systems based on stimulus-responsive materials are very demanding for controlled and long-term drug release in pulsed or continuous manner by stimulating externally through a noninvasive process. It will be very beneficial also if release is possible under the action of an external stimulus in noninvasive way because this type of method will give a new avenue for the treatment of many disease which require daily invasive intervention. We are interested to use magnetic field responsive technique as it is minimally invasive and magnetic material as targeting agent to send it to the specific region of interest in the body by applying external magnetic field. On the other hand, heat dissipation by the magnetic particles under AC magnetic field can be controlled by controlling applied magnetic field from outside the body and normal tissues can be saved. To achieve this control, we need to study the phenomena with many possible magnetic materials.

Before the use of the materials in such fields, their physical properties should be investigated properly with change of many parameters to have an overall idea about them so that it can be tuned according to requirements. Over the past few years scientific investigation on hollow nano sphere materials with high stability, large surface area, high porosity have drawn special interest. Among them hollow magnetic materials have drawn significant interest for the use in the field of water purification, biomedical application etc.^23^,^24^. Previously, we also have synthesized hollow like magnetite particles by PVP method and measured magnetic properties^25^. The PVP method successfully produced only magnetite hollow particles but not other kinds of ferrites. In this paper, a method has been described for synthesis of nano hollow sphere magnetite of different sizes by oleylamine micelles templated way. By applying this oleylamine technique, many other kinds of ferrites can also be synthesized. Among them some are published and some are under investigation^26^. Therefore, to develop a general method for synthesis of many kinds of hollow like ferrite particles, we have concentrated on this method and also received success to some extent. On the other hand, PVP being a very large molecule in presence of ethylene glycol, produce a sticky substance thereby causing the process not easy to be handled. Our interest is in use of nanoparticles for drug release and theraputic agents. To make it a reality, a comparative study is needed to be made with various kinds of ferrites of similar group. We will be able to synthesize various kinds of ferrites by the same procedure which will be very useful for comparative study and to do an optimization in the use of materials and techniques. We are interested in hollow like particles because in case of hollow sphere, the added advantage is that due to hollow shape of particles, more amounts of drugs can be loaded. Applying AC magnetic field from outside the body, temperature of particles can be controlled, which in turn will provide a control over drug release from the drug loaded particles. Applying AC magnetic field with different frequency and strength in either pulsed or continuous application techniques, the control over drug release can be achieved. Our method in this respect will be highly potential if it can be materialized in real world. But before its practice it is necessary to be investigated systematically.

## Experimental details

All the chemicals such as Ferric chloride hexahydrate (FeCl_3_, 6H_2_O), ethylene glycol (EG), alcohol, urea and oleylamine are of analytical grade and purchased from Sigma–Aldrich.

The synthesis procedure of the hollow spheres is as follows: Here three different sets of particles were prepared by varying the amount of oleylamine micelles. 2.4 gm of FeCl_3_, 6H_2_O was mixed in 40 ml Ethylene Glycol (EG) and 20 ml ethyl alcohol solution for all sets of particles. This solution was stirred with a magnetic stirrer until the Ferric chloride was completely miscible in the solution. Then 1.06 gm of Urea and different amount of oleylamine were added in the above solution to prepare different sets of particles. These solutions were again stirred until it became completely transparent. These final solutions were then transferred in three different 80 ml teflon lined stainless steel autoclaves for the solvothermal process. The solvothermal process was carried out at 180 °C for 15 hrs. After 15 hrs, resultant black solutions were washed in alcohol for several times and then dried at 60 °C overnight. Particles were prepared in three different amount of oleylamine such as 2 ml, 3 ml and 4 ml to see the effect of change of concentration of oleylamine and to understand the mechanism of formation of NHS.

Here, role of Ethylene Glycol (EG) is to reduce the Fe^+3^ ions to Fe^+2^ ions partially. Urea helps to precipitate Fe^+3^ and Fe^+2^ to their corresponding hydroxides where the hydroxyl group comes from ammonium-hydroxide produced from urea and after the heat treatment both the iron hydroxides forms Fe_3_O_4_ through removal of water. The possible chemical reaction is given below what is shown in our previous published result^27^.


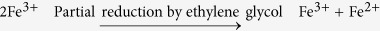



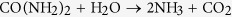



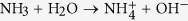



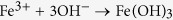



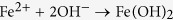



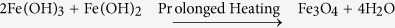


### Drug loading experiment

50 mg of prepared NHS is dispersed in 1 ml of deionized water then 50 μL of ammonium hydroxide solution is added to this and stirred for 30 min then 100 μL of aqueous 1.5 × 10^−3^ M DOX solution is added under stirring condition and stirring is continued for one hour to allow the drug to be intercalated in the nanoparticles. Then the drug loaded particles are separated out through centrifugation technique and then dried. The dried particles are then gently washed with de-ionized water to remove excess non-bound drug. The amount of DOX loaded in this system is calculated from the absorbance value obtained from UV-visible absorption spectra at ~500 nm of initial DOX solution and solution collected after separating the particles. Loading efficiency is measured by applying the equation proposed by a group Nigam *et al*.^28^. and obtained in case of 350 nm NHS is near about 70%.

### Characterization techniques

The phase and morphology of the synthesized samples were characterized by X–ray diffraction, scanning electron microscope (SEM) and transmission electron microscopy (TEM). The XRD analysis was performed in Rigaku Miniflex II desktop X–ray diffractometer using Cu K_α_ (λ = 1.5418 Å) source. The TEM investigations of the hollow spheres were done by TECHNAI G^2^ SF20 ST TEM operating at 200 kV. The frequency dependent AC hysteresis loops of all the samples were measured at room temperature (25 °C) using our lab-made instrument.

## Results and Discussion

### Crystallography and Morphology

**Figure 1:**
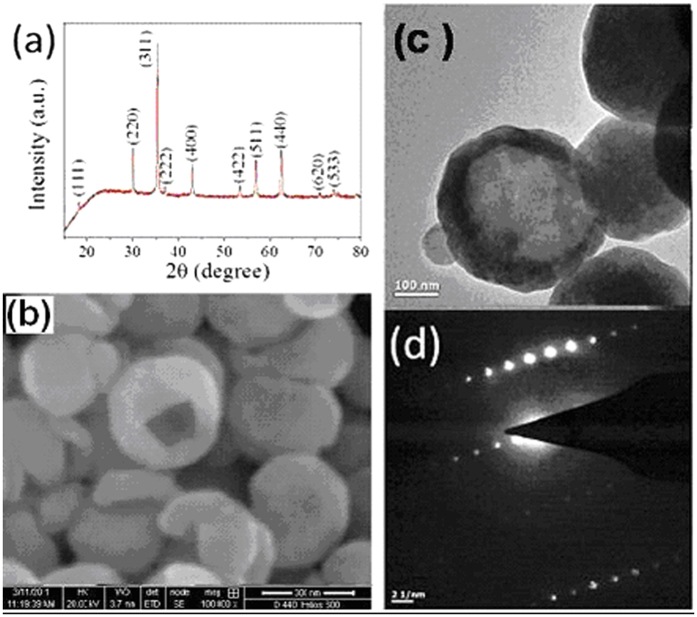
X-rd spectrum (**a**), SEM (**b**) and TEM (**c**) micrographs and SAED pattern (**d**) of the magnetite hollow spheres of diameter ~350 nm.

**Figure 2:**
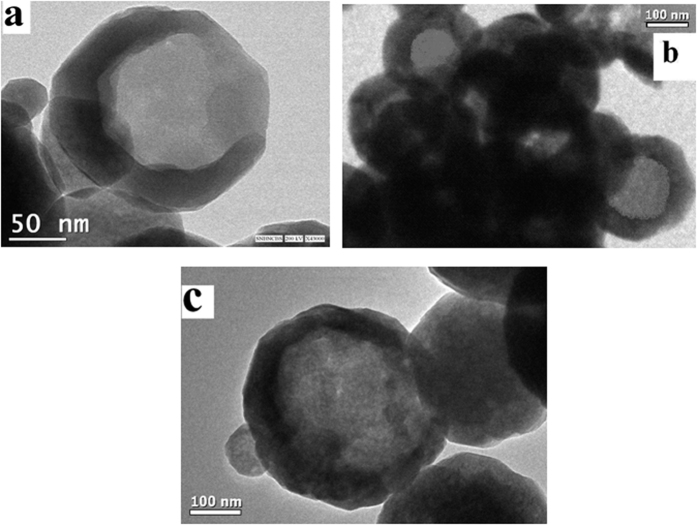
TEM images of three different sized particles prepared in three different amount of oleylamine: (**a**) is prepared in 4 ml, (**b**) is in 3 ml and (**c**) is in 2 ml of oleylamine.

Figure 1 shows the measurements done on one of the samples (highest size) to elucidate the crystal structure and morphology of the as synthesized material. The X-rd pattern of one set of the samples is shown in Fig. 1(a) and indexed as well. All the diffraction peaks of the pattern can be indexed to the face centred cubic (fcc) structure of magnetite (Fe_3_O_4_) [JCPDS card no. 65-3107]. The sharp diffraction peaks indicate higher crystallite size of the Fe_3_O_4_ samples. The crystallite size (*d*) of the sample was calculated using the Debye Scherrer equation (*d* = 0.9λ/(*β*cos*θ*)). The crystallite size (d) of the sample was calculated taking into account the most intense peak and found to be ~24 nm. Lattice parameter was calculated considering the XRD peaks 511, 440, and 533, which come at 2θ value of 56.93, 62.57 and 74.1078 respectively. The high angle peaks are considered here to minimize the error. The calculated lattice parameter obtained are 8.404, 8.397, and 8.389 A° for XRD peaks 511, 440, and 533 respectively. Taking the average of these lattice parameters we get the value 8.39666 A°, which matches with the lattice parameter for magnetite. Hence particles are of pure phase magnetite. The SEM micrograph (Fig. 1(b)) of the sample shows hollow nature of spherical magnetite. Average diameter of the hollow spheres after considering the SEM micrographs is found to be nearly 350 nm. Distinct hollow structure of the as synthesized Fe_3_O_4_ spheres is confirmed from the TEM micrograph as shown in Fig. 1(c). The selected area electron diffraction (SAED) pattern taken from the complete hollow sphere (Fig. 1(d)) exhibits the high crystalline nature of the Fe_3_O_4_ NHS. TEM images at three different concentrations of oleylamine are shown in Fig. 2. Figure 2a presents the image of sample prepared in 4 ml, 2b is prepared in 3 ml and 2c is prepared in 2 ml oleylamine. Here we see with decrease of concentration of oleylamine, particle size increases and shell thickness also increases. Changes in different size parameters like size of whole particles, crystallite size and shell thickness of the hollow like particles with change of amount of oleylamine (from 4 to 2 ml) are shown in Table 1.

### Hollow sphere formation mechanism

**Figure 3:**
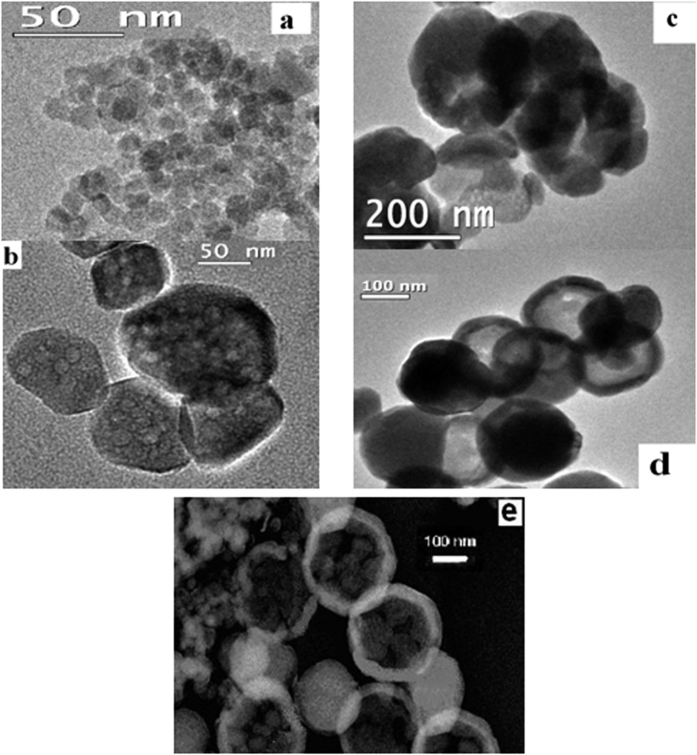
TEM images of time dependent synthesis of particles (**a**) after 2 h, (**b**) after 5 h (**c**) after 10 h and (**d**) is after 15 h, (**e**) dark field TEM image of NHSs.

**Figure 4:**
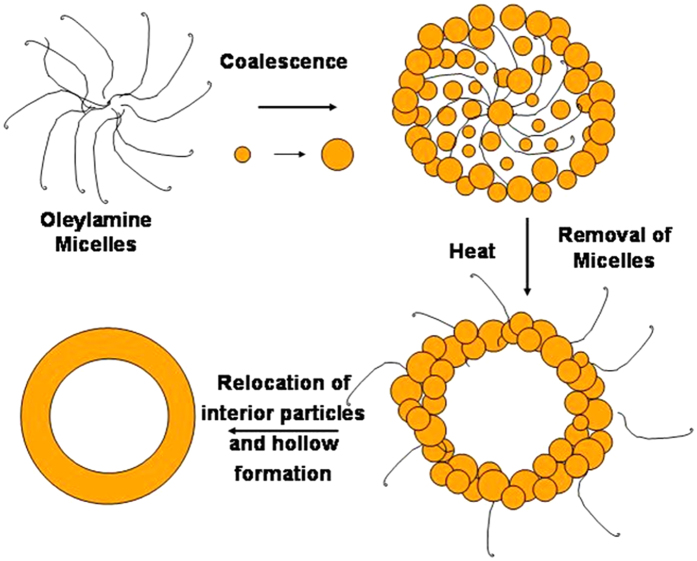
Schematic diagram of formation mechanism of hollow sphere particles by oleylamine micelles.

In order to find out the progress of hollow formation in our sample, we have collected an aliquot at different time interval (2, 5, 10, and 15 h) during synthesis of sample, followed by washing and drying procedures and investigated those particles through TEM analysis. A series of TEM images (as shown in Fig. 3(a–d)) of the resulting products indicate increase in size of the particles and a distinct change in surface and interior of the nano-spheres with time. Initially, after reaction of 2 h, smaller nano structures are observed (as shown in Fig. 3(a)) and these small nano-particles are found to be aggregated to form a porous sphere with rough surface (as shown in Fig. 3(b)) during 2–5 h interval. With a longer reaction time after 10 h, a hollowing effect is started (as shown in Fig. 3(c)) to be formed from the porous spheres. After 15 h of reaction time, the distinct contrast between the edge and the hollow interior of the spheres (as shown in Fig. 3(d)) confirms the formation of nano hollow spheres (NHSs). From the above described TEM images, it appears that at the beginning, nano-scale hydroxides of iron nucleate then coalesce to each other and simultaneously deposit on the surface of micelles formed by oleylamine, thus the smaller particles grow to larger with time, driven by Ostwald ripening process^29^. Another possibility is that micelles accompanying smaller particles come closer and grow to larger one to decrease the surface energy. Then with prolonged heat treatment the molecules, ‘oleylamine’ which form micelles, move out towards edge from the centre of the larger particles through process of evaporation. During this evaporation process a force is created from centre to outward direction that is toward edge from the core or centre of the bigger particles. Due to this outward directed force during evaporation process the smaller particles situated in the core of the larger particles tend to be relocated at the edge of the bigger one which leads to nano-hollow spheres formation. Here we see with increase of concentration of oleylamine thinner shell is formed. It can be explained by the phenomenon that with increase of concentration of oleylamine, more number of micelles are formed and with increase of number of micelles, deposition of smaller particles on the surface of micelles become lesser which leads to thinner shell formation as in each case precursor salt concentrations of Fe are the same. One of the representative dark field TEM images of NHS is shown in Fig. 3e, which again confirms the above explained phenomenon as few of smaller particles still are visible here at the core of NHSs. The underlying mechanism for the formation of NHSs is more clearly shown by the schematic diagram (Fig. 4).

**Figure 5:**
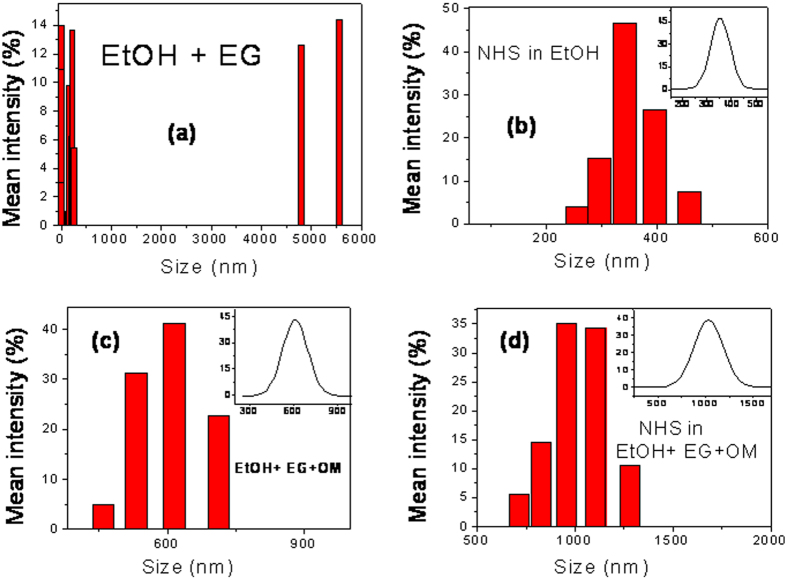
Dynamic light scattering study on different sets of solution including OM and excluding OM. (**a**) representative histogram on size distribution obtained from DLS measurement for solution consisting of ethanol (EtOH) and ethylene glycol (EG), (**b**) representative DLS measurement on solution consisting of NHS dispersed in ethanol (EtOH), (**c**) histogram and spectrum obtained from DLS study on solution consisting of ethanol (EtOH), ethylene glycol (EG) and oleylamine (OM), (**d**) histogram and spectrum obtained from DLS measurement on solution consisting of ethanol (EtOH), ethylene glycol (EG) and oleylamine (OM) and NHSs.

To prove the oleylamine based micelles formation the dynamic light scattering (DLS) study was performed for different solutions including as well as excluding oleylamine (OM) as shown in Fig. 5(a–d). The measurement was performed for 350 nm NHS. Figure 5(a) represent DLS study on solution consisting of ethanol (EtOH) and ethylene glycol (EG). Here the size shown is not significant. It may be due to very small amount of dust particles present in solution and some impurities. Figure 5(b) represents DLS study on solution consisting of NHS dispersed in ethanol (EtOH). This indicates size of NHS present in solution. Figure 5(c) represents DLS study on solution consisting of ethanol (EtOH), ethylene glycol (EG) and oleylamine (OM). Here it is evident from the size that the OM forms micelles. After addition of NHS in this solution the size become larger. It is because formation of micelles is dynamic in nature and after addition of NHS the OM micelles take NHS inside the core expanding to larger size.

### FTIR measurements

**Figure 6:**
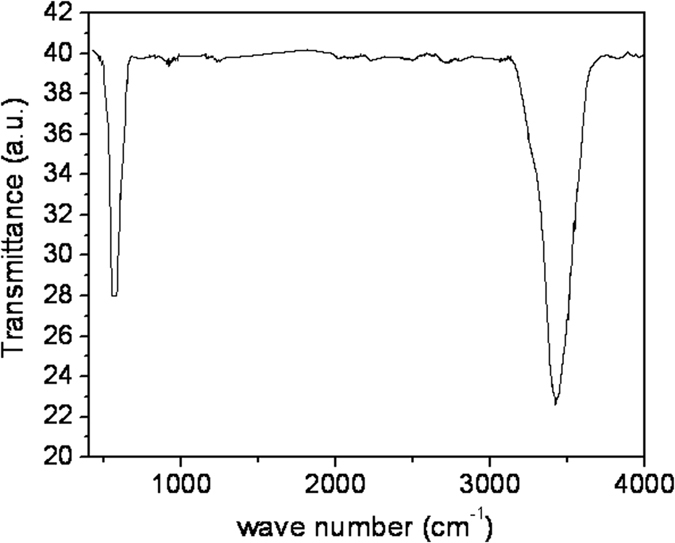
FTIR spectrum taken for one batch of NHSs in KBr matrix method.

FTIR measurement was performed for one batch of samples on KBr matrix and is shown in Fig. 6. We see here a broaden peak at about 465 to 686 cm^−1^, which is attributed to Fe-O-Fe bending vibration of magnetite. Hence particles are of magnetite^30^,^31^. One of the groups^28^ has again shown that a peak in FTIR spectrum at 580 cm^−1^ is attributed to Fe-O stretching vibration of magnetite. In our case the peak lie in the region which is not beyond 580 cm^−1^. Hence this result again supports the magnetite phase of NHSs.

### Magnetic measurements and drug release studies

**Figure 7:**
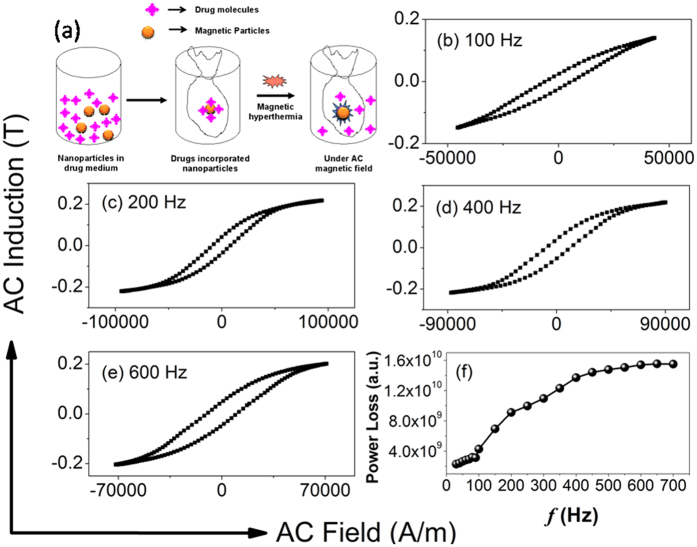
(**a**) represent schematically drug release under AC magnetic field, (**b–d**) present hysteresis loop measurements at different frequencies and (**e**) is frequency dependent loss power measurement for 200 nm particles.

**Figure 8:**
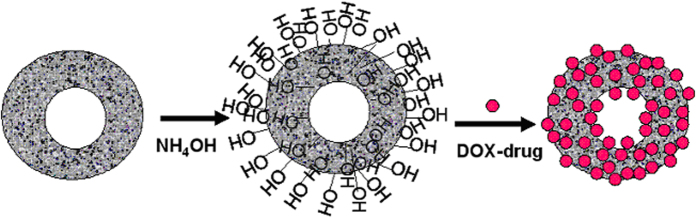
Schematic diagram on drug loading through intercalation of drug molecules to NHSs.

**Figure 9:**
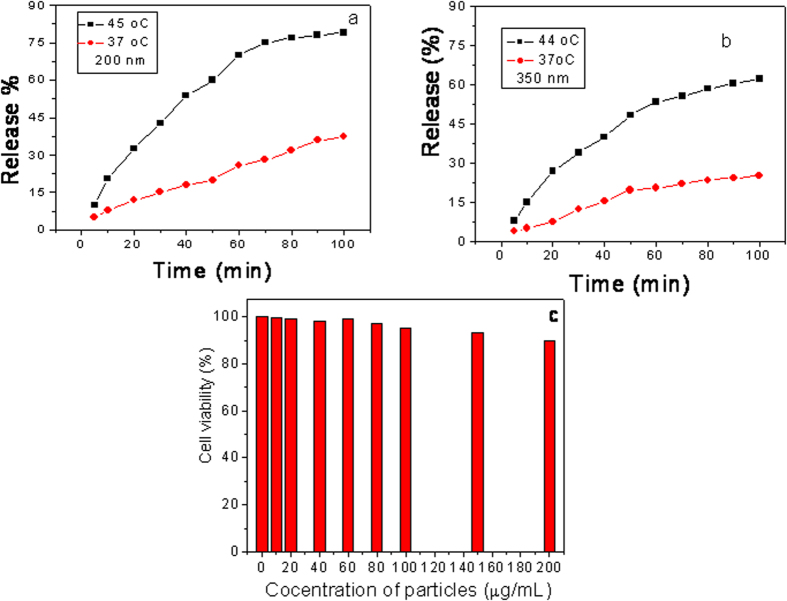
AC magnetic field stimulated drug release study for two sets of drug loaded particles (**a**) for 200 nm and (**b**) for 350 nm and (**c**) is the graph of reflection on *in vitro* cytotoxicity of free NHS of 200 nm size on Hella cell line.

We are interested to use the particles for drug release by hyperthermia technique applying AC magnetic field as shown schematically in Fig. 7(a). Therefore we have done some AC magnetic measurements on these hollow spheres in our lab made set-up where we can produce nearly 92 kA/m ac magnetic field at nearly 700 Hz AC field frequency. The AC hysteresis loops of the hollow spheres were measured from 30 Hz to 700 Hz AC field frequency and the corresponding loops for four different frequencies for 200 nm particles are shown in Fig. 7(b–e). From these figures it is clear that the coercivity of the hysteresis loops of the hollow spheres increases with increasing frequency of AC field. Actually depending on frequency of AC magnetic field, particles can either behave superparamagnetically or ferromagnetically. At lower frequencies, few particles having relaxation time less than the measuring time will behave superparamagnetically. Most of the particles will behave ferromagnetically when the measuring time is less than the relaxation time of the particles, i.e. at higher frequencies. So, as the frequency of the AC magnetic field increases, the fraction of ferromagnetic particles increases and this phenomenon results higher coercivity at higher frequencies. The variation of power loss (proportional to the product of hysteresis loop area and frequency) with frequency is shown in Fig. 7(f). Power loss is obtained integrating the hysteresis loop area and multiplying it with measurement frequency^32^,^33^,^34^. From the power loss calculation, an idea about the conversion of electromagnetic energy to heat energy by these particles are obtained. It is known that higher the power loss higher will be the heat energy generated by the particles. From the Fig. 7(f) it is reflected that the power loss of the hollow sphere increases with increasing frequency of applied AC field. We are able to measure the loss power up to 700 Hz AC field in our set up because after that the impedance of the circuit becomes so high that the field is reduced and reduced field is not enough to saturate the hysteresis loops. But with this limitation we measured the temperatures of the solutions of dispersed magnetic particles applying the AC magnetic field at different frequencies. From these results we observed that when 10 mg of particles is dissolved in 3 ml of solution, the temperature of the solution become 44 °C for 350 nm NHS at applied AC frequency 700 Hz and for 200 nm NHS at applied AC frequency of 550 Hz. At the 44 °C temperature we studied the drug release activity for two sets of particles 200 nm and 350 nm. 10 mg of drug loaded particles were taken in 3 ml PBS buffer of pH 7.4. Intercalation of drug (DOX) with porous hollow particles is shown schematically in Fig. 8. After functionalization with ammonium hydroxide, particles are hydroxylated as shown in this schematic diagram. Then drugs are allowed to be loaded into this hydroxide functionalized particles. After drying these drug loaded particles through removal of H_2_O from hydroxylated particles and the drug molecules, the drugs are intercalated into the particles in a similar way as shown by the group Parrot *et al*.^35^. The drug release event at 37 °C and under AC magnetic field that is at 44 °C was studied at different interval of time. The representative UV-visible spectra on drug release activity for two sets of particles at 37 °C and at 44 °C are shown in Fig. 9(a,b). From the Fig. 9(a,b) it is observed that under AC magnetic field drug is released with faster rate. It is may be due to doxorubicin drug is heat sensitive and under AC magnetic field particles are heated up and release the drug with faster rate. From this figure it is also observed that for smaller particles drug release rate is higher than the bigger particles. One reason is may be due to the fact that the effective surface to volume ratio for smaller particles is higher than that of the bigger particles. Our lab made instrument have some limitations. Due to lack of funding we can not modify the instrument. But with all the limitations from the preliminary results we obtain the information regarding AC magnetic field stimulated drug release will be a new avenue for cancer treatment in a better way. In such case lower amount of drugs will be more effective and will act in a better way as control over drug release is possible according to required drug dose by changing different parameters of magnetic particles as well as applied AC magnetic fields. Thus in this method large options are possible to be opened up for the release of drugs in a control manner. It will be more cost effective as well as will produce lower side effects also. We have checked the cell viability test using our free nano hollow sphere (NHS) of 200 nm size before drug loading. Stability of particles in cell culture medium has also been studied. We kept particles in cell culture medium at 44 °C for one day then separated the particles by centrifuge method and dried, then XRD measurement was done which gave same pattern as was observed for as made particles. This result indicates magnetite particles are stable in cell culture medium for sufficient amount of time. *In vitro* cytotoxicity of the free i.e. without drug loaded NHS on Hella cell line is studied by varying the amount of NHS. The Hella cell line were cultured in incubator at 37 °C with 5% CO_2_ in RPMI 1640 media with 10% fetal calf serum (FCS) in 5 cm culture dishes. Culture at 80% confluence were routinely split 1:10 in culture dishes following general PBS and Trypsin method. Then 100 mg of magnetite particles were dispersed in 2 ml of water by probe sonication method. Different aliquot from this magnetite dispersion was added to cell culture dishes maintaining the final concentration from 10 to 200 μg/mL and kept for 24 hours in incubator. One control experiment was also done. When the magnetite was taken 10 μg/mL, the cell viability was almost 99% and gradually decreases very slowly with increase of particles concentration which is reflected in Fig. 9(c). During experiment we also see that almost no morphological changes of the cells take place before and after treatment with particles. These results indicate that the cytotoxicity of our particles is very low for high pharmacological efficiency as this dose is enough for a cell. Hence our particles will be much more compatible for body as they are almost non-toxic upto very high dose. Hence these results indicate that our particles qualitatively better in respect of other results^36^,^37^.

## Conclusion

Magnetite hollow sphere particles have been prepared using oleylamine micelles using as core substance and varying the number of formation of micelles, size and shell thickness of the particles also have been altered successfully. The mechanism of evolution of hollow in the magnetite particles by solvo-thermal synthesis method has also been revealed here by support of some experiments and logical arguments. Structural characterization of these particles shows that they are pure phase of magnetite. The AC magnetic field stimulated drug release activity gives us new information about better treatment pathway for cancer and other diseases where control release in a noninvasive way is required. Hence these particles will be useful for drug release sensitizing the particles by hyperthermia technique applying an altering AC magnetic field from outside the body.

## Additional Information

**How to cite this article**: Goswami, M. M. Synthesis of Micelles Guided Magnetite (Fe_3_O_4_) Hollow Spheres and their application for AC Magnetic Field Responsive Drug Release. *Sci. Rep.* **6**, 35721; doi: 10.1038/srep35721 (2016).

**Publisher’s note:** Springer Nature remains neutral with regard to jurisdictional claims in published maps and institutional affiliations.

## Figures and Tables

**Table 1:** Change of different size parameters of three sets of particles with change of amount of oleylamine.

Amount of oleylamine	Size of the particles	Shell-thickness	Crystallite size
2 ml	350 ± 5 nm	~80	~24 nm
3 ml	280 ± 5 nm	~60	~22 nm
4 ml	200 ± 5 nm	~35	~21 nm
